# What do atomic bomb survivors teach us about therapy-free remission in people with chronic myeloid leukaemia?

**DOI:** 10.1038/s41375-023-02081-x

**Published:** 2023-11-10

**Authors:** Tomas Radivoyevitch, Robert Peter Gale, Matt E. Kalaycio

**Affiliations:** 1 Department of Quantitative Health Sciences, Cleveland Clinic, Cleveland, OH USA; 2 Centre for Haematology, Department of Immunology and Inflammation, Imperial College of Science, Technology and Medicine, London, UK; 3 Department of Hematology and Medical Oncology, Cleveland Clinic, Cleveland, OH USA

**Keywords:** Epidemiology, Chronic myeloid leukaemia

## To the Editor:

People with chronic myeloid leukaemia (CML) who stop tyrosine kinase inhibitors (TKIs) and are in therapy-free remission (TFR) can have minimal residual disease (MRD) [1]. One explanation is that anti-CML immune responses control CML clone growth [2]. This is controversial [3]. If true, too few leukaemia stem cells (LSCs) and their progeny may fail to stimulate immunity and too many may overwhelm it [4].

**Fig. 1:**
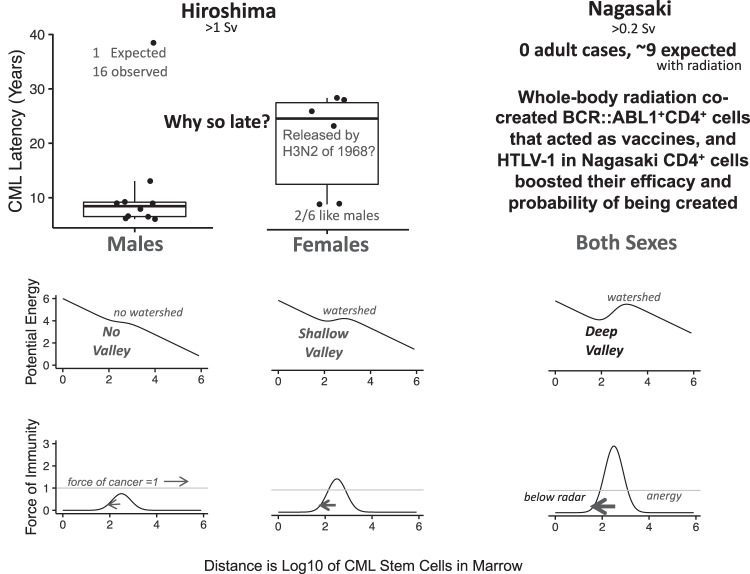
CML in Japanese atomic bomb survivors. In Hiroshima, there were so few survivors of >1 Sv that many years of follow-up were needed to expect 1 case of CML had there been no radiation. The male case nearly 40 years after the bombing is thus age-induced and the remaining 15 cases are all radiation-induced. Four Hiroshima female cases were very delayed, consistent with stronger immunity in females, and clustered in onset years, consistent with being released from immunity via H3N2. Nine Nagasaki cases expected based on Hiroshima rates were absent. Bottom plots depict how immunity forces can impact energy landscapes and thus clone size stability; animated versions of these plots (Supplemental Fig. S1) show how H3N2-mediated reductions in force of immunity peak heights could have clustered Hiroshima onsets.

The A-bombings of 1945 generated two mysteries [5]. One is in Hiroshima radiation-induced CML occurred with expected latencies of 5–10 years in 2 of 6 females and in 9 of 10 males, but in 4 of 6 females, onsets were delayed and clustered from 1969 to 1974. The other is in Nagasaki survivors no cases of CML were observed in a dose cohort in which ~9 were expected (Fig. 1).

Why were 4 Hiroshima female radiation-induced cases delayed and why were their onsets clustered? Delays may have occurred because immunity is more likely to be induced in females who have stronger immune responses compared with males [6]. Clustering may have occurred because the 1968 Hong Kong flu pandemic entered Japan in 1969, the same year as the first delayed female Hiroshima CML onset case, and evolved into the H3N2 seasonal flu [7]. Put otherwise, we hypothesise that H3N2 exhausted anti-CML immunity in the A-bomb survivors and that this released latent neoplastic radiation-induced *BCR::ABL1*-positive cells and thus clustered delayed onset Hiroshima females [8]. A study of mice provides some support for this idea [9].

Why was immunity in Nagasaki so strong that onsets were delayed indefinitely in both sexes? Hiroshima-Nagasaki city differences in CML (Fig. 1) may have occurred via endogenous HTLV-1 infection present in Nagasaki but not in Hiroshima [10]. This hypothesis has 3 contingencies: (1) CD4-positive cells expanded in numbers before the bombings because of endemic HTLV-1 infection and were the target of radiation-induced *BCR::ABL1*-positive non-neoplastic clones; (2) numbers of such cells were sufficiently high such that every adult exposed to >0.2 Sieverts (Sv) in Nagasaki developed ≥1 CD4-positive cell with a *BCR::ABL1* translocation and consequently developed immunity to CML including CML unassociated with radiation exposure; and (3) relative to Hiroshima, CD4-positive cells were more effective in both sexes in Nagasaki because there they expressed not only *BCR::ABL1* but also HTLV-1-infection-related genes. Put otherwise, we hypothesise CD4-positive T-cells delivered *BCR::ABL1* immunogenic peptides to invoke immunity, HTLV-1 was an adjuvant that boosted anti-CML immunity, and CD4-positive, *BCR::ABL1*-positive clones were self-limiting by acting as auto-vaccines [8].

**Fig. 2:**
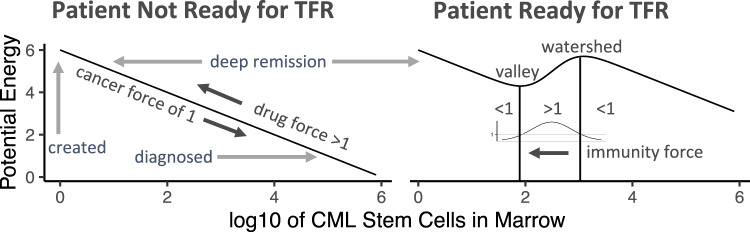
Force-energy view of immuno-control. Initially, (left) patient energy landscapes may not have a valley, but with time one may arise (right) and enable a TFR. Energy here is the integral of net force times distance in log10 units of LSC numbers. The net force is the sum of a cancer force of 1 pushing right and a drug force of >1 (if it is effective) pushing left. If immunity exists (right), it also pushes left; here its force is a Gaussian with a mean of 2.5 and a standard deviation of $1/\sqrt{2\pi }$. As long as the force of immunity peak is >1, TFR by immunity is reachable. If so a valley arises wherein LSC numbers could stably exist.

A study of people attempting TFRs claims to have identified 3 types of subjects [4]. We suggest these cohorts correspond to people who are Hiroshima-male-like (incapable of holding a TFR), Hiroshima-female-like (capable of holding a TFR but at random times of immuno-suppression this TFR can be lost), and Nagasaki-like (capable of holding TFRs robustly across immuno-compromising perturbations) [8]. To further connect A-bomb survivors to these TFR cohorts we focus on the founding LSC, which is likely a very primitive, TKI-resistant cell [11]. We suggest that if someone with CML lives sufficiently long this founding LSC will produce a *BCR::ABL1*-positive CD4-positive T-cell which will form a benign clone that will protect him/her against neoplastic LSCs. The existence of this immune-enhancing LSC founder event is consistent with *BCR::ABL1* expression in T-cells in 11 of 20 subjects with CML in TFR [12]. It is also consistent with CD4-positive T-cells expanding because of high expression of *ABL1* [13]. An immune-enhancing event such as the LSC founder creating a CD4-positive T-cell could create an energy landscape valley in which *BCR::ABL1* transcripts could stably exist (Fig. 2). Thus, beyond teaching us that TFR MRD states could arise via energy landscape valleys, A-bomb survivors teach us to look for new ways to control CML via CD4-positive T-cells; new ways are still needed, at least in the US [14].

In a study of 68 patients attempting TFRs, 5 who succeeded had MRD, perhaps via immuno-control, and 39 who succeeded had undetectable disease, perhaps via eradication [1]. Thus, as low as 11% (5/44) of TFRs may have been by immuno-control. Of these, half might be Nagasaki-like and half Hiroshima-female-like [4, 8]. Only the latter (~5% of TFRs) are expected to be susceptible to relapses via flu or COVID infections.

Ten of 10 CML cases among Chernobyl accident cleanup workers arose after long delays [15]. As they were all young when exposed, the delays could reflect 20–30 years being needed to reach ages at which CML is more common. Without an estimate of the number of CML cases expected if Chernobyl accident cleanup workers were not exposed, it is unclear how many of the 10, if any, were Hiroshima-female-like.

Onset clustering in Hiroshima and cases missing in Nagasaki are hard to explain [5]. Evidence for our explanation includes the alternatives having inconsistencies [8]. Via our explanation, A-bomb survivors teach us to look for a CD4-positive cell role in CML immunity.

### Supplementary information

Supplemental Figure S1

## Data Availability

A-bomb survivor data is publicly available from RERF.
