# Supplementary material for: What do atomic bomb survivors teach us about therapy-free remission in people with chronic myeloid leukaemia?
Source: Leukemia. 2023 Nov 10;38(1):207–9. doi: 10.1038/s41375-023-02081-x (PMC10776394; doi:10.1038/s41375-023-02081-x)
Supplement: Supplementary file 1 — Supplemental Figure S1 [file 41375_2023_2081_MOESM1_ESM.pdf]

Supplemental Figure S1 is a Powerpoint presentation with animation. Please download it from https://zenodo.org/records/10014700 and run it as a slide show in Powerpoint.
